# Applying machine learning methods for the analysis of two-dimensional mass spectra

**DOI:** 10.1140/epja/s10050-023-01080-x

**Published:** 2023-07-25

**Authors:** Z. Gao, A. Solders, A. Al-Adili, O. Beliuskina, T. Eronen, A. Kankainen, M. Lantz, I. D. Moore, D. A. Nesterenko, H. Penttilä, S. Pomp, H. Sjöstrand, the IGISOL team

**Affiliations:** 1 Department of Physics and Astronomy, Uppsala University, BOX 516, 75120 Uppsala, Sweden; 2 Department of Physics, Accelerator laboratory, University of Jyväskylä, P.O. Box 35(YFL), 40014 Jyväskylä, Finland

## Abstract

In a measurement of isomeric yield-ratios in fission, the Phase-Imaging Ion-Cyclotron-Resonance technique, which projects the radial motions of ions in the Penning trap (JYFLTRAP) onto a position-sensitive micro-channel plate detector, has been applied. To obtain the yield ratio, that is the relative population of two states of an isomer pair, a novel analysis procedure has been developed to determine the number of detected ions in each state, as well as corrections for the detector efficiency and decay losses. In order to determine the population of the states in cases where their mass difference is too small to reach full separation, a Bayesian Gaussian Mixture model was implemented. The position-dependent efficiency of the micro-channel plate detector was calibrated by mapping it with $^{133}$Cs$^+$ ions, and a Gaussian Process was trained with the position data to construct an efficiency function that could be used to correct the recorded distributions. The obtained numbers of counts of excited and ground-state ions were used to derive the isomeric yield ratio, taking into account decay losses as well as feeding from precursors.

## Introduction

The isomeric yield ratio (IYR) of fission products, referring to the relative independent yield of one of the isomeric states to the total independent yield of that nuclide, is important for the modeling of the fission process. The relative yields of the states also affect nuclear reactor operation and decay heat calculations, and certain isomeric yield ratios are important in reactor based studies of the antineutrino mixing angle, $\theta _{13}$ [1, 2]. Another example is the modeling of the r-process in stellar nucleosynthesis [3, 4] where some isomers, due to their unusually long lifetime, play an important role.

Penning Trap Mass Spectrometry (PTMS) is a versatile tool to study fundamental properties of matter via high-precision atomic mass measurements, utilizing the relationship between the mass (*m*), charge (*q*) and cyclotron frequency ($\nu _c$) of an ion in the magnetic field (*B*) of the trap:

1 $$\begin{aligned} \nu _c = \frac{1}{2\pi }\frac{q}{m}B. \end{aligned}$$

One method to determine the cyclotron frequency is the so-called Phase-Imaging Ion-Cyclotron-Resonance (PI-ICR) technique [5, 6]. Due to the presence of an electric field in the trap, the cyclotron motion is split into two radial motions with characteristic frequencies, the reduced cyclotron frequency ($\nu _+$) and the magnetron frequency ($\nu _-$):

2 $$\begin{aligned} \nu _c = \nu _+ + \nu _-. \end{aligned}$$

The two frequencies can be determined from their respective phase ($\phi _\pm$) acquired in a set accumulation time, by extracting the ions from the trap and projecting their positions onto a position-sensitive Micro-Channel Plate (MCP) detector.

With the application of the PI-ICR technique [7] in the double Penning trap JYFLTRAP [8, 9] at the University of Jyväskylä, a mass resolution of a few ten keV has been demonstrated for medium heavy ions (at $\textit{A}$ = 130), using an accumulation time of 320 ms [10]. Together with the capability of the Ion Guide Isotope Separator On-Line (IGISOL) facility to produce fission fragments via particle-induced fission [11], this provides an opportunity to measure IYRs through direct ion counting of mass-separated fission fragments. By allowing isomers of the same nuclide to revolve in the trap with the mass-dependent reduced-cyclotron frequency, the ions will become phase-separated by the ratio of mass over charge. After extraction and detection on the MCP the number of counts in each state can be determined.

Mass-separating techniques have been employed to measure fission yields for many decades, but not until the introduction of Penning Traps has it been possible to separate isomers by mass and determine the yields by direct ion counting. Instead, other techniques, for example radiochemical method and $\gamma$ spectroscopy [12–14], have been employed to determine IYRs. However, the nuclides that are accessible with those techniques are limited by, for example, the availability of decay schemes, the half-lives, and the yields. With the PI-ICR technique, the number of accessible nuclides are increased since no knowledge of decay schemes are needed (except for second-order decay corrections) and nuclides with half-lives down to a few hundred milliseconds can be studied [10]. Furthermore, it has been successfully demonstrated that IYR of fission products with yields as low as 0.006% can be measured with this technique [15].

In the previous measurement of IYR with the PI-ICR technique [15], the analyses of the phase images were performed by fitting the angular distributions with Gaussian functions. However, this procedure has proven to be unreliable in cases where the ion species are not well separated. Here we present an alternative technique, where a Bayesian Gaussian Mixture (BGM) model, as implemented in the Sci-Kit package [16], was trained on the data to obtain the number of nuclei in each state.

Furthermore, to account for the position-dependent efficiency of the MCP, a dedicated measurement of the efficiency was performed and modeled using a Gaussian Process [18] (GP) that was used to correct the extracted count rates.

Based on timing information, the IYRs of the fission products were analytically derived from the obtained number of ions on the MCP by considering decay losses and feeding from precursors.

## Measurement procedure

A 25-MeV proton beam with a current of up to 10 $\mu$A from the K-130 cyclotron was used to induce fission in a 15 mg/cm$^2$ natural uranium target in the IGISOL [19] fission ion-guide. The fission products that emerged from the target were thermalized in a helium buffer gas at a pressure of 300 mbar and extracted from the ion guide with the gas flow. After post acceleration in the Sextupole Ion Guide SPIG [11], the ionized fission products were transported to the downstream dipole magnet for mass-to-charge ($\textit{A/q}$) selection and then collected in the radio-frequency quadrupole (RFQ) cooler and buncher [20]. From the RFQ, short bunches of cold ions were extracted and transported to JYFLTRAP for trapping and identification [7–9].

The typical time from a fission event until the detection of the mass-separated fission products on the MCP after JYFLTRAP, including preparation and separation in the Penning trap, ranges from a few hundred milliseconds to a few seconds. In some cases, this time is comparable to the half-life of either of the studied states and/or their precursor. In these cases, the decay losses and feeding have to be accounted for.

The beam-lines and relevant techniques for mass measurements have been thoroughly described in previous publications [7–9, 11, 15, 20, 21]. Here only a brief description will be presented in order to outline how the time spent in each stage was estimated for the decay correction.

**Thermalization** The thermalization of the fission products in the helium gas is several orders of magnitude faster than the transportation of the ions out of the gas cell with the gas flow. Therefore, this time can safely be neglected in the decay correction.

**Drifting** After the thermalization, the fission products are carried by the gas flow toward the exit of the ion guide. The average ion drifting time is estimated to be about 100 ms [21].

**Transport** The fission products are extracted from the ion guide by a sextupole ion guide (SPIG) [11] and accelerated to 30q keV (where q is the charge state of the fission product) for further transport to a dipole magnet for isobar selection. This transportation is fast (tens of microseconds) and can be neglected in the decay correction.

**RFQ** After the isobar selection in the dipole magnet, the ions are injected into a gas-filled RFQ cooler and buncher. The RFQ is filled with ions at a constant rate for a time defined by the opening and closing of a potential set by the electrodes of the cooler. In order to avoid space charge effects in the Penning traps [21], the opening time of the RFQ is set to obtain a suitable number of ions per bunch, and hence depends on the fission rate and yield of the nuclei of interest. Typically, the filling takes up to a few hundred milliseconds.

**Trap 1** In the next step, the bunched ions are transported to the purification trap of JYFLTRAP where the nuclei of interest are separated from the rest of the isobar chain by employing the sideband cooling technique [7, 8, 22]. The purification typically also takes a few hundred milliseconds.

**Table 1:** The time the ions spend in each stage of the measurement cycle. $t_{acc}$ is the phase accumulation time which is part of the time the ions spend in Trap 2

Ion	t$_{acc}$	Drifting	RFQ	Trap 1	Trap 2
	ms	ms	ms	ms	ms
$^{129}$In$^+$	99	100	200	260	227
	157	100	260	260	284
$^{129}$Sn$^+$	1010	100	35	260	1267
$^{129}$Sb$^+$	24	100	10	260	152
	39	100	10	260	167
	39	100	10	260	167

**Trap 2** In the precision trap of JYFLTRAP the ground state and the long-lived excited state (s) are separated by the mass-to-charge ratio. Before that, a second cleaning, which takes about 120 ms, is performed to remove any remaining isobars. The separation is then obtained by allowing the ions to freely rotate with the mass-dependent reduced cyclotron frequency until they are separated by a phase $\varDelta \phi$ which is determined by

3 $$\begin{aligned} \varDelta \phi = 2\pi t_{acc} \varDelta \nu , \end{aligned}$$

where $t_{acc}$ is the so-called phase-accumulation time, and $\varDelta \nu$ is the difference in reduced cyclotron frequencies of the two states. The phase-accumulation time is preferably set to obtain a maximum phase difference of $\pi$ radians. Typical values in this experiment range between tens of milliseconds up to more than one second. From the phase-accumulation times the corresponding phase separations are predicted and presented in Table 3 together with corresponding observed separations. Additionally, a 2 ms conversion pulse is applied in the trap to convert the cyclotron motion into the slow magnetron motion before the ions are ejected from the trap and their positions projected onto the MCP detector [7].

Table 1 summarises the time the ions of interest in this work spend in each stage of the measurement cycle.

**Fig. 1:**
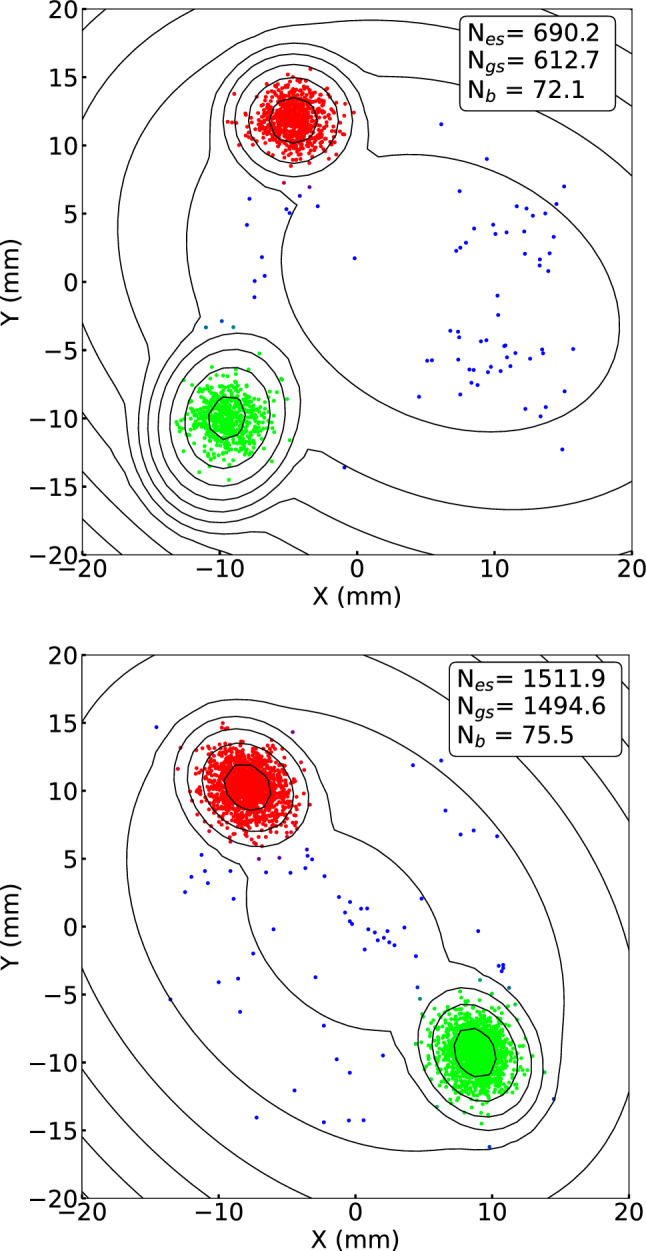
Typical phase images of the reduced cyclotron motion for $^{129}$Sb$^+$ ions obtained with the PI-ICR technique. The color mixture of each point represents the probabilities of belonging to the excited state (red), the ground state (green), and the background (blue). The determined number of nuclei in each component from the phase image is estimated by the BGM model. In the upper panel the phase-accumulation time is set to 24 ms, while in the lower panel it is 39 ms. Contour lines illustrate the normal distributions of the components of the BGM model

Figure 1 shows examples of phase images of the mass spectra of the fission product $^{129}$Sb$^+$, for which the excited state has an excitation energy of 1924.3 keV and a half-life of 17.7 min [23]. In order to eliminate a small background of ionized rest gas molecules, a gate on the ions’ flight time from Trap 2 to the MCP detector was used. This TOF gate was set to ensure that all ions of interest are kept.

To demonstrate how the phase difference depends on the phase accumulation time, two different times, 24 ms and 39 ms, are shown. Since the direction of the cyclotron motion is known to be counterclockwise, this allows identification of the two states. In this case, the green spots in Fig. 1 represent the ground state of $^{129}$Sb$^+$, while the red spots represent the excited state. Visible in the images are also counts in blue. These are believed to be contaminant ions leaking from trap 1 or ions from either state that for some reason lost their phase. These events are treated as background in the analysis.

## Data analysis

### Fitting of the angular distribution

**Fig. 2:**
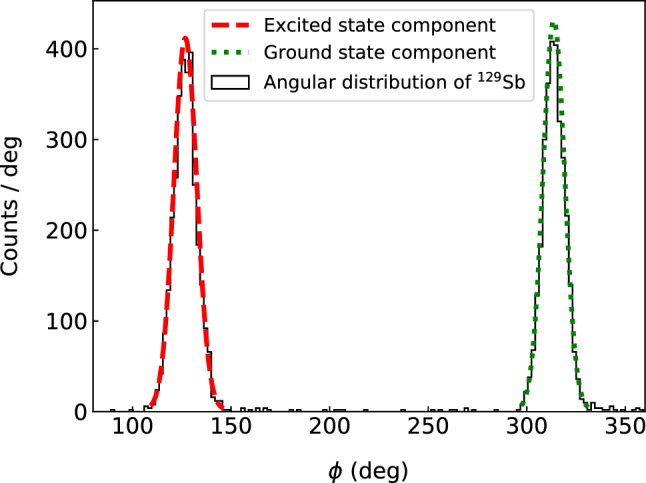
Histogram in the polar coordinate for $^{129}$Sb$^+$ ions with a phase accumulation time of 30 ms and fitting of the angular distribution with a double normal distribution

In the case of $^{129}$Sb$^+$ ions, due to the relatively high excitation energy, the long-lived excited state is well separated from the ground state even for a short phase accumulation time. In cases like these, the count rates of the two states could be determined simply by selecting a region around each center. However, to better account for the background, and to be able to determine the number of counts also when the states overlap, the analysis has previously been performed by transforming the data into polar coordinates and fitting the angular distributions with two (or more) normal distributions [15], see Fig. 2.

**Fig. 3:**
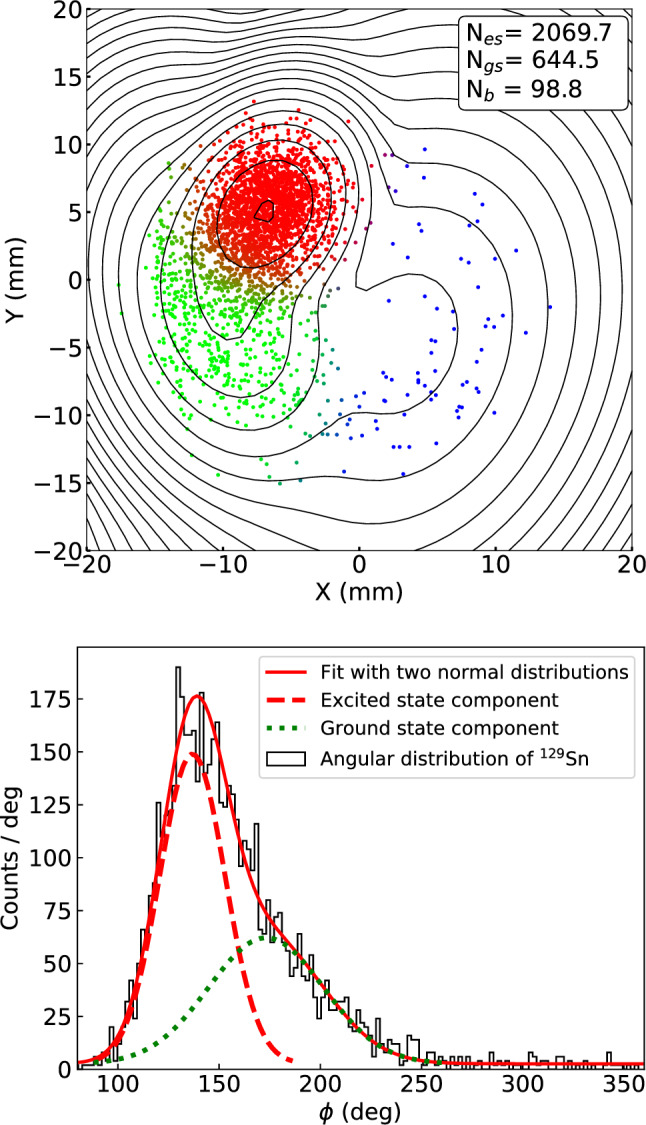
Upper panel: The phase image of the reduced cyclotron motion for $^{129}$Sn$^+$ ions obtained with a phase accumulation time of 1010 ms. The color mixture of each point represents the probabilities of belonging to the excited state (red), the ground state (green), or the background (blue). The determined number of nuclei in each component from the phase image is estimated by the BGM model. Contour lines represent the normal distributions of the components of the BGM model. Lower panel: The angular distribution of the data. The solid red curve shows a least square fit of the angular distribution with a double normal distribution. The dashed lines show the excited state and the ground state component of the fitted distribution

However, in some cases, the excitation energies are so small that this method of separation becomes unreliable. An example is $^{129}$Sn$^+$ ions for which the energy difference between the excited state and the ground state is 35.15 keV [23]. As a demonstration, the method of separation by angular fitting was performed for both $^{129}$Sb$^+$ and $^{129}$Sn$^+$. The lower panel of Fig. 3 shows the result of the fitting for $^{129}$Sn$^+$. In this case, the two states are not separated even for a phase accumulation time of 1010 ms.

In the separation by angular fitting the radial information is disregarded. Gating on the radii could in principle be an additional constraint but, considering that there are very few background events in the phase image, such a gate would have a negligible impact on the angular distribution.

**Fig. 4:**
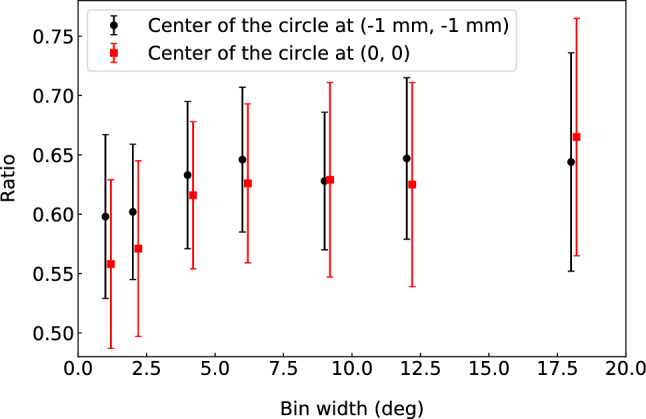
Ratios of $^{129}$Sn that are obtained from the angular fittings to the histograms with different bin widths and centers

Furthermore, it has been observed that the selection of bin width of the histogram, as well as the determination of the center for the coordinate transformation, could have a significant impact on the fitting, and subsequently on the determined number of counts. While the center could in principle be determined through a dedicated measurement, the binning problem is more difficult to address. Figure 4 shows the calculated ratios from angular fits to histograms with different bin widths and centers. As seen in the figure, the choice of bin widths and center positions have significant impacts on the determination of the IYR.

### Bayesian Gaussian Mixture (BGM) model

As an alternative to the fitting of the angular distribution, a method to separate the two states based on BGM has been developed. BGM is a variant of the Gaussian mixture model, which models the data points with a mixture of a finite number of Gaussian distributions, to which a variational inference algorithm is added [16]. In the initialization of the model, hyperparameters, such as the number of components, the convergence threshold, and the maximum number of iterations, are set. For example, in the case of $^{129}$Sn$^+$ the component number was set to 3, the convergence threshold to $10^{-12}$, and the maximum number of iterations to 1000. Then the initial model is trained with the position data. After the training, three components: the excited state (es), the ground state (gs), and the background (b), with amplitudes significantly greater than zero were obtained from the model. Increasing the number of components results in components with amplitudes close to zero, demonstrating that the data is best described by three components. From the model, information on the amplitude, the center position, and the covariance matrix of each component could be obtained. The possibility to use the Gaussian Mixture Model to determine the center positions of the event clusters for mass measurements has been demonstrated by Weber et al. [17]. The Gaussian distribution of each component is reflected by the contour lines in Fig. 1 and in the upper panel of Fig. 3.

The BGM model also estimates the probabilities of each data point belonging to either components: $P_{es}$, $P_{gs}$ and $P_b$. In Fig. 1 and in the upper panel of Fig. 3, these probabilities are indicated by a color mixture using the RGB color system. Thus, the number of nuclei in the excited state ($N_{es}$) can be estimated from the sum of probabilities $P_{es}$. In the same way, the number of nuclei in the ground state ($N_{gs}$) and the background ($N_{b}$) is estimated from $P_{gs}$ and $P_b$.

To estimate the uncertainty in the number of counts, the statistical method Bootstrapping [24], which samples from a replacement raw data set, has been applied 10,000 times. By applying the BGM model on the 10,000 samples, the standard deviations of $N_{es}$ and $N_{gs}$ were obtained.

### Comparison of the methods

In Table 2, the ratios of the excited state over the total, as obtained from the angular fits and the BGM model before applying any correction, are presented. In the case of $^{129}$In the population of the excited state is much lower than that of the ground state. Considering that the fission product $^{129}$In has a low fission yield, very few events of the excited state were recorded. In cases like this, where one state is much less populated, the procedure of fitting the angular distribution becomes sensitive to the binning of the histogram. This is reflected by the larger-than-expected deviation between the two results for the angular fit. The same discrepancy is not observed using the BGM model. In the case of $^{129}$Sb, for which the ratio is close to 0.5 and the excited state is well separated from the ground state, the two analysis methods agree within uncertainties.

For $^{129}$Sn, where the two peaks are overlapping, the angular fitting becomes sensitive to the determination of the center of the polar system, as well as to the angular binning. In cases like these, the BGM model seems to give more reproducible results, as training the model on the $^{129}$Sn data with different hyper-parameters results in the same converged model.

**Table 2:** Ratios of the counts of the excited state over the total counts in the cases of $^{129}$In$^+$, $^{129}$Sn$^+$, and $^{129}$Sb$^+$. The ratios were calculated from the fits to the angular distributions and from the BGM model, respectively

Nuclide	$t_{acc}$ (ms)	Ratios from	Ratios from
		angular fit	BGM
$^{129}$In	99	0.117 (8)	0.208 (18)
	157	0.167 (5)	0.185 (8)
$^{129}$Sn	1010	0.58 (7)	0.757 (20)
$^{129}$Sb	24	0.532 (9)	0.529 (10)
	39	0.503 (5)	0.504 (7)
	39	0.610 (6)	0.606 (7)

### Homogeneity of the MCP

**Fig. 5:**
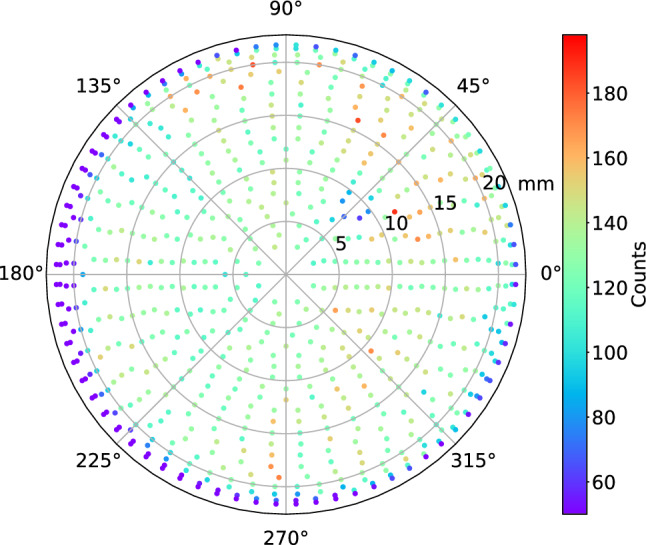
Two-dimensional histogram of the number of counts in a calibration run with $^{133}$Cs$^+$-ions

Before the population of the states can be determined the relative detection efficiency at different positions of the MCP detector has to be corrected for. To obtain this, the surface of the MCP detector was scanned with $^{133}$Cs$^+$-ions in a calibration measurement. Figure 5 shows the number of counts per bin at each beam position.

**Fig. 6:**
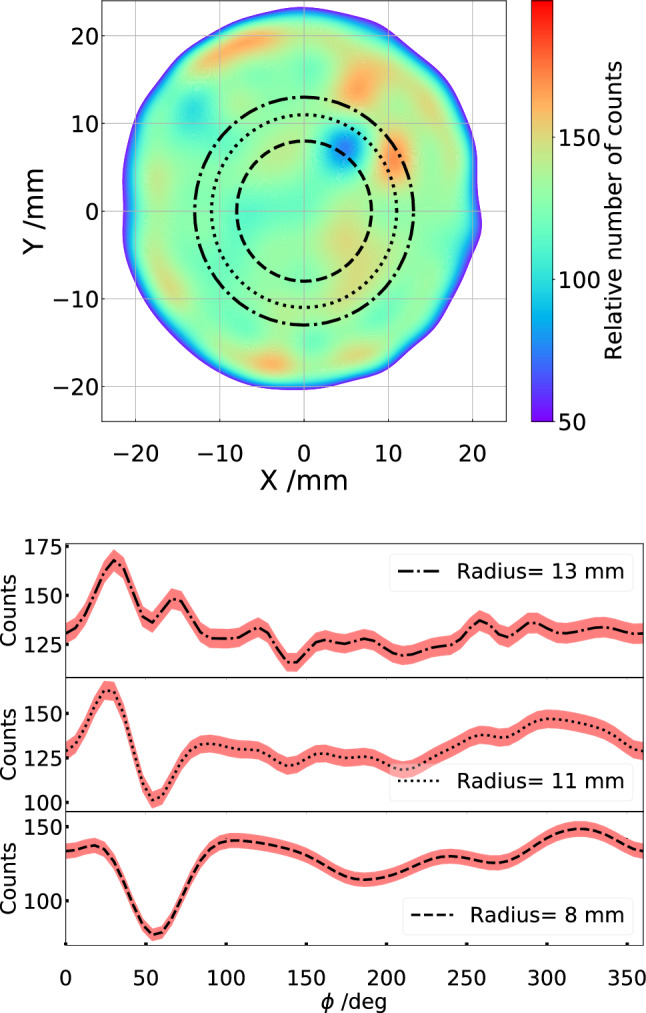
The top panel shows the relative detection efficiency as modeled with a GP. The lower panels show the predicted relative efficiencies with uncertainties at 95% confidence level for the radii 13 mm, 11 mm, and 8 mm, respectively

Based on this, the count distribution is modeled by a Gaussian Process [18] (GP) using the corresponding module from Sci-Kit [16]. The top panel of Fig. 6 shows the predicted number of counts, which represent the relative efficiency, at different positions. The lower panels of Fig. 6 present angular distributions of the predicted number of counts for the radii 13, 11, and 8 mm. As can be observed, the detection efficiency is not homogeneous and has to be corrected for in the data analysis.

An area with significantly lower efficiency can be noticed around x = 5 mm, y = 8 mm in the upper panel of Fig. 6. The area corresponds to the significant drop of the relative number of counts around 50 $\deg$ in the bottom panel of Fig. 6. This is the result of radiation damage to the detector due to an extended exposure time.

To correct for the variation in the efficiency of the MCP, every event in the Bootstrap sampling was

weighted by

4 $$\begin{aligned} w_i= \frac{1}{\epsilon _i}, \end{aligned}$$

where $\epsilon _i$ is the relative efficiency at the corresponding position. To account for the uncertainty in the efficiency, $\epsilon _i$ was sampled from a normal distribution with mean and standard deviation obtained from the GP.

The result of the Bootstrapping procedure is 10,000 efficiency-corrected samples of the population of the states. The mean and standard deviation of the number of nuclei in each state from the 10,000 samples are hence the best estimates of the efficiency-corrected number of events.

As a consistency check, rather than weighting the points during the sampling with Bootstrapping, the weights were instead included in the training of the BGM model. Both methods give the same results, including uncertainties.

### Decay correction

In some cases, such as for $^{129}$Sb, where the half-lives of the long-lived excited state, the ground state and the precursor are much longer than a measurement cycle, the isomeric yield ratio could be directly calculated from the efficiency-corrected number of counts at the MCP:

5 $$\begin{aligned} R \equiv \frac{Y_{es}}{Y_{es}+Y_{gs}}=\frac{N_{es}}{N_{es}+N_{gs}}, \end{aligned}$$

where $Y_{es}$ and $Y_{gs}$ are the fission yield of the excited state and the ground state, respectively.

In other cases, for example $^{129}$In and $^{129}$Sn, the measured numbers of nuclei have to be corrected for the decay of the fission products and/or the feeding from the precursors before the isomeric yield ratio can be calculated.

**Fig. 7:**
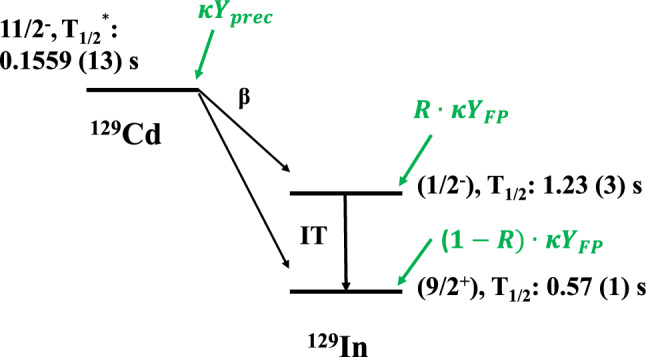
Part of the decay scheme of $^{129}$Cd with the isomers of $^{129}$In. $T^{*}_{1/2}$ is the combined $\beta$ decay half-life of the ground state and the excited state of $^{129}$Cd [27]. $\kappa Y$ represents the direct production rate from the ion guide without taking decays into account

To estimate the impact the decay of the fission products has on the isomeric yield ratio in every step of the measurement, a simplified decay scheme, as shown in Fig. 7, is used. Here, the spins, parities and half-lives from the literature [23, 25–27] are marked beside each level. $^{129}$In also has two excited states at 1650 keV (23/2$^-$) and 1941 keV (29/2$^+$) [23] with halflives longer than 100 ms that should, in principle, be included in the correction. However, their impact on the yield ratio between the excited state 1/2$^-$ and the ground state was estimated to be negligible, and are therefore not presented in Fig. 7. The ground state and the excited state of $^{129}$Cd are not clearly identified in the ENSDF database [25]. Hence, the combined $\beta$ decay half-life of both states was adopted from the most recent measurement [27]. Since all other states have much shorter half-lives they are assumed to decay instantly.

In the following, the combined effects in the ion guide; including transportation efficiency in the target, stopping efficiency in the helium gas, loss due to collisions with the walls, and so on, are assumed to be the same for all nuclei of the same isobar [28]. Due to charge exchange interactions between the fission products and impurities of the helium gas in the ion guide, element-dependent variations in the charge-state distribution of the extracted ions are expected [21]. This so-called chemical effect is not fully understood; however, it does not affect the isomeric yield ratio directly. Instead, it appears as a possible second-order contribution to the decay correction. Because of this, and for simplicity, the chemical effect is ignored in the analysis of the yield data. All nuclide-independent losses can be combined into a constant $\kappa$, so that the production rate of any nuclei (ignoring decays) can be written $\kappa Y$, where *Y* is the fission product yield of interest.

**Drifting** For any nuclide, taking the decay loss into account during the drifting through the buffer gas in the ion guide, the corresponding production rate is governed by:

6 $$\begin{aligned} P = \kappa Y e^{-\lambda t_{drift}}, \end{aligned}$$

in which *Y* is either the fission yield of the fission product of interest $Y_{\textrm{FP}}$ or the cumulative yield of its precursor $Y_{\textrm{prec}}$. $\lambda$ is the decay constant, and $t_{drift}$ is the duration of the drifting.

In addition to the direct production from fission, any nuclide that is the daughter of a long-lived state gets fed via decay. The production governed by the decay is described by:

7 $$\begin{aligned} \frac{dP_{d}}{dt} = \lambda _{p} P_{p} (t)-\lambda _{d} P_d (t), \end{aligned}$$

where the first term, consisting of the decay constant $\lambda _p$ and the production rate $P_p$ of the parent nuclide, describes the feeding from the parent nuclide while the second term accounts for the decay losses by multiplying the decay constant $\lambda _d$ with the time-dependent production rate $P_d$ of the daughter nuclide.

Substituting $P_{p}$ in Eq. (7) with the expression in Eq. (6), the general solution of the feeding and decay effect on the production of the daughter nuclide can be expressed as:

8 $$\begin{aligned} P_d = \kappa Y_p \lambda _p \frac{e^{-\lambda _d t_{drift}}-e^{-\lambda _p t_{drift}}}{\lambda _p - \lambda _d} \equiv \kappa Y_{p} \cdot D_{p2d}, \end{aligned}$$

where the parent-to-daughter feeding factor $D_{p2d}$ has been introduced. Depending on the decay mode, the feeding factor will describe either the $\beta$-decay ($\beta$) or isomeric transition (*IT*). It is worth noticing that Eq. (8) is similar to the well-known Bateman equation [29].

From Fig. 7, the production rate of the excited state of $^{129}$In can be expresses as:

9 $$\begin{aligned} P_{es} = R \cdot \kappa Y_{\textrm{FP}} e^{-\lambda _{es}t_{drift}} + \beta \cdot \kappa Y_{\textrm{prec}} D_{\beta }^{es}, \end{aligned}$$

where the first term represents the production directly from fission, *R* is the to-be-derived isomeric yield ratio, and $Y_{\textrm{FP}} = Y_{\textrm{es}} + Y_{\textrm{gs}}$ is the total yield of the two states of the fission product of interest, i.e. $^{129}$In. The second term, in which $Y_{\textrm{prec}}$ is the accumulative yield of precursor $^{129}$Cd and $\beta$ is the $\beta$-decay branching ratio of $^{129}$Cd to $^{129m}$In, represents the contribution from the decay of $^{129}$Cd.

If available, the value of $\beta$ is taken from the ENSDF database [25]. If not, it is instead uniformly sampled from an interval based on the branching ratios of $\beta$ decays of neighboring nuclei.

For the ground state of Indium, taking both the decays from the precursor ($^{129}$Cd) and from the excited state ($^{129m}$In) into account, the result is:

10 $$\begin{aligned} P_{gs}= & {} (1-R) \cdot \kappa Y_{FP} e^{-\lambda _{gs}t_{drift}} + (1-\beta ) \kappa Y_{prec} D_{\beta }^{gs} \nonumber \\{} & {} + IT \cdot \kappa Y_{es} D_{IT}^{gs}. \end{aligned}$$

Here $D_{\beta }^{gs}$ is the feeding factor of $^{129}$Cd to the ground state of $^{129}$In. *IT* is the branching ratio of the isomeric transition from the excited state as obtained from ENSDF [25], thus $D_{IT}^{gs}$ is the feeding factor of the isomeric transition from $^{129m}$In. The second-order effect of the decay-chain feeding from the precursor via the excited state to the ground state has been neglected.

**RFQ** The RFQ is filled with ions at a constant rate which,

assuming the transportation efficiency from the ion guide to the RFQ is the same for all fission products, is the production rate (*P*) obtained in the previous step.

In general, when no feeding from precursors is considered, the accumulated number of nuclei of a radioactive nuclide N is calculated by integrating the filling rate over the opening time of the RFQ gate $t_{RFQ}$.

11 $$\begin{aligned} N = P \int ^{t_{RFQ}} e^{-\lambda (t_{RFQ}-t)}dt = P D_{RFQ}, \end{aligned}$$

where $D_{RFQ}$ is the decay factor that represents the integral decay effect over the filling time. For instance, Eq. (11) is applied to calculate the accumulated number of nuclei of $^{129}$Cd.

For a daughter nucleus with zero filling rate, that is only fed by the decay of the precursor, the accumulation of the number of nuclei $N_d$ is described by the equation:

12 $$\begin{aligned} \frac{dN_d}{dt} = \lambda _{p}N_{p} (t) - \lambda _d N_d (t), \end{aligned}$$

where $N_{p} (t)$ is the time-dependent number of the parent nuclide.

By inserting the time-dependent number of the precursors governed by Eq. (11) into Eq. (12), the solution of the accumulated number of the daughter nuclei is:

13 $$\begin{aligned} N_d = P_{p}\left[ \frac{1-e^{-\lambda _{d}t_{RFQ}}}{\lambda _{d}}-\frac{e^{-\lambda _{d}t_{RFQ}}-e^ {-\lambda _{p}t_{RFQ}}}{\lambda _{p}-\lambda _{d}}\right] = F_{p2d}P_{p}, \nonumber \\ \end{aligned}$$

where $F_{p2d}$ represents the parent-to-daughter factor of which $_{p2d}$ will be replaced by $\beta$ or *IT* once the decay mode is specified.

In the case of $^{129m}$In, the accumulated number of nuclei in one bunch is obtained by summing the number obtained from Eq. (11) with those obtained from the decay of $^{129}$Cd which is governed by Eq. (13),

14 $$\begin{aligned} N^{RFQ}_{es} = P_{es} D_{RFQ} + \beta F^{es}_{\beta } P_{prec}, \end{aligned}$$

where $F^{es}_{\beta }$ is the feeding factor of the $\beta$ decay of $^{129}$Cd to $^{129m}$In. Similarly, in the case of the ground state of $^{129}$In, the number of nuclei in one bunch $N^{RFQ}_{gs}$ is obtained from the filling rate $P_{gs}$, the decay of the precursor $^{129}$Cd, and the decay of the excited state $^{129m}$In to the ground state.

The feeding factor of the decay-chain from the precursor via the excited state to the ground state is ignored also here.

It is worth noticing that the decay factor and the parent-to-daughter factor in Eqs. (11) and (13) are constant when the filling time is known. This means that the number of nuclei in the bunch is a linear combination of the obtained production rates and hence linear with respect to the fission yields.

**Trap 1** In the purification trap, when no precursor is considered, the number of radioactive nuclei $N^{T1}$ is described in a general way by:

15 $$\begin{aligned} N^{T1} = N^{RFQ} e^{-\lambda t_{T1}}, \end{aligned}$$

where $N^{RFQ}$ is the number of nuclei in a bunch transported from the RFQ and $t_{T1}$ is the duration in trap 1.

The integrated feeding effect to a daughter nucleus from a parent nucleus in the trap is governed by the well-known Bateman equation [29]:

16 $$\begin{aligned} N^{T1}_{d} = \lambda _{p}N^{RFQ}_{p}\frac{e^{-\lambda _{d}t_{T1}}-e^{-\lambda _{p}t_{T1}}}{\lambda _{p}-\lambda _{d}} = N^{RFQ}_{p} P^{T1}_{p2d}, \end{aligned}$$

where $P^{T1}_{p2d}$ is the parent-to-daughter feeding factor for a decay. When $t_{T1}$ is fixed, the decay factor in Eq. (15), as well as the parent-to-daughter feeding factor $P^{T1}_{p2d}$ in Eq. (16), is constant.

By applying Eq. (15) and Eq. (16) to the excited state and the ground state of the fission product of interest, their numbers in trap 1, $N^{T1}_{es}$ and $N^{T1}_{gs}$, could be calculated.

**Trap 2** During the second cleaning in trap 2, the feeding from the excited state to the ground state is taken into account using Eq. (16). After that, it is assumed that the nuclei at the excited state and the ground state would start separating in a short time. Thus only the decay losses of the states were taken into account:

17 $$\begin{aligned} N^{T2} = N^{T1} e^{-\lambda t_{T2}}, \end{aligned}$$

where $$t_{T2}$$ is the duration in trap 2 and $$N^{T2} $$ is the number of counts of each state when ions are being ejected.

Due to the time of flight of ions from trap 2 to the MCP detector being about 45 $\mu$s, the decay loss in this process is negligible. The calculated number of nuclei in trap 2 equals the determined numbers from the phase image on the MCP detector $N^{MCP}$ which was corrected for detector efficiency.

18 $$\begin{aligned}{} & {} N^{T2}_{es} = N^{MCP}_{es} \end{aligned}$$

19 $$\begin{aligned}{} & {} N^{T2}_{gs} = N^{MCP}_{gs} \end{aligned}$$

Combining equations from (6) to (19) results in two equations with three unknown variables: the cumulative yield of the precursor ($Y_{prec}$), the summed fission yield of the excited state and the ground state ($Y_{FP}$), and the to-be-derived isomeric yield ratio (*R*).

If $Y_{\textrm{prec}}$ is replaced with $\eta Y_{\textrm{FP}}$, where $\eta$ is the ratio of the cumulative yield of the precursor over the summed yield of the measured fission product, the system can be solved analytically to obtain the isomeric yield ratio, *R*. The value of $\eta$ is calculated from the fission yields obtained from the fission model GEF 2023/1.2 [30]. The fission yields from GEF were assumed to have an uncertainty of 5% and hence $\eta$ is assumed to have an uncertainty of 7%.

Since the combined equations from (6) to (19) are too complex to do error propagation, the uncertainty estimation of the isomeric yield ratio was conducted by sampling the parameters and repeating the calculations 10,000 times.

The numbers of counts of states were sampled from normal distributions with means and standard deviations as obtained in section 3.4. All parameters with available uncertainties, for example the half-lives and $\eta$, were also sampled from normal distributions. When the branching ratio is not available directly, its value was sampled uniformly from an interval based on the incomplete decay scheme and the $\beta$ decays of neighboring nuclei in ENSDF [25]. The drifting time was uniformly sampled from an interval [50, 150] ms. Applying every set of sampled parameters to the equations, 10,000 isomeric yield ratios were obtained. From this, the mean and the standard deviation were adopted as the isomeric yield ratio and the corresponding uncertainty.

**Fig. 8:**
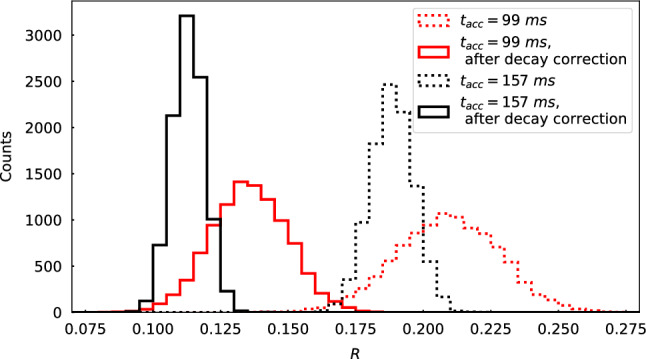
Dotted histograms show the ratios of the determined number of counts of $^{129}$In before applying the decay correction for the measurements with the accumulation time of 99 ms (red) and 157 ms (black). Solid-line histograms show the IYRs considering the decay loss and feeding effect due to radioactive decays

Figure 8 presents histograms of the obtained ratios for $^{129}$In with two different accumulation times. The dotted lines show the ratios before applying the decay correction, while the solid lines are the corrected IYR. In the decay scheme of Fig. 7, the branching ratio of the isomeric transition of $^{129m}$In is below 0.3% [25], and the branching ratios from $^{129}$Cd to the excited state and the ground state of $^{129}$In are 32 (12)% and 68 (12)% [31]. Considering the average $\eta$ is 0.01 which means the feeding from $^{129}$Cd is negligible, the decay losses of the isomer and the ground state of $^{129}$In are the dominant contributions to the decay correction. Thus, it makes sense that the ratios decreased significantly after the decay correction because the excited state of $^{129}$In has a longer half-life than the ground state.

From Fig. 8, it can be observed that the absolute uncertainties of the ratios are reduced after the decay correction. However, the relative uncertainties increased from 4 % to 5 % and 9 % to 10 % since more sources of uncertainty were included.

It is worth mentioning that the decay scheme used for the correction becomes more complicated when more long-lived states have to be taken into account. For example, in the decay correction for $^{129}$Sn both the $^{129}$Cd and $^{129}$In decay schemes, as well as the derived isomeric yield ratio of $^{129}$In, have to be included. This means more terms have to be added in Eqs. (6)–(19), while the number of unknown variables stay the same.

## Results and discussion

Table 3 presents the corrected isomeric yield ratios of $^{129}$In, $^{129}$Sn, and $^{129}$Sb from different measurements. Compared to the values obtained before the correction (Table 2) the differences between the derived values for the same nuclide are reduced, an indication that the corrections are going in the right direction. However, for $^{129}$Sb the obtained ratio from the third measurement still does not agree with the two others within uncertainties. This measurement was performed with the positions of the states rotated 180$^{\circ }$ compared to the second. The reason for the discrepancy is therefore likely to be an insufficient efficiency correction or unknown systematic uncertainties in the calibration. To some extent, the discrepancy has been addressed by making the PI-ICR measurements at two orientations, which allows an averaging of the two. However, in future measurements, more orientations could be added in order to even out any variation in the efficiency. Also, if possible a more homogeneous MCP should be used and more effort should be put into the efficiency calibration.

**Table 3:** Results of the isomeric yield ratios (IYR) of $^{129}$In, $^{129}$Sn, and $^{129}$Sb from different measurements. Relevant information on the fission products from the literature and the settings of the measurements are included. The literature data are from Ref. [23] unless stated otherwise. The determined angular separation of the states is also listed and compared to the value predicted based on the literature mass values [23, 32]

Nuclide	Ground state		Excited state			IYR	$t_{acc}$	Determined	Predicted
	$T_{1/2}$	Spin	Energy	$T_{1/2}$	Spin	from	(ms)	separation	separation
			(keV)			this work		(deg)	(deg)
$^{129}$In	0.57 (1) s [26]	(9/2$^{+}$)	447 (13) [32]	1.23 (3) s	(1/2$^{-}$)	0.136 (14)	99	104.6 (92)	110.6 (32)
						0.113 (6)	157	178.3 (5)	175.4 (52)
$^{129}$Sn	2.23 (4) m	3/2$^{+}$	35.15 (5)	6.9 (1) m	11/2$^{-}$	0.777 (20)	1010	51.5 (42)	88.7 (4)
$^{129}$Sb	4.366 (26) h	7/2$^{+}$	1851.31 (6)	17.7 (1) m	(19/2$^{-}$)	0.514 (10)	24	118.8 (3)	111.0 (1)
						0.515 (7)	39	189.7 (2)	180.4 (1)
						0.576 (7)	39	189.2 (2)	180.4 (1)

From the obtained center positions of the components of the BGM model, the angular separation between the states is obtained and listed in Table 3. Predicted separations calculated from the mass ratios and the phase accumulation times approximately agree with the observed angular separation, except in the case of $^{129}$Sn. In this case, the small relative mass difference between the two states makes the required phase accumulation time larger than one second. This is already close to the typical upper limit of the phase accumulation time of about 1.2 s [33], which could explain the discrepancy. Other possible explanations include the space charge effect, imperfection of the magnetic and/or electric fields of the Penning trap, and incomplete conversion from the cyclotron motion to the magnetron motion before the extraction. All these effects have been identified as possible sources of uncertainties in PI-ICR mass measurements [33].

## Conclusions

A complete analysis routine, from the analysis of the phase image to the estimation of decays of fission products, for determining the isomeric yield ratio has been developed. In this process, machine learning methods were applied to analyse the phase images obtained with the PI-ICR technique. The BGM model shows excellent capability in modeling scatter points in two dimensions with little prior information. By automating the procedure, manual biases, for example bin width selection and center of the polar coordinate system used in the angular distribution analysis, are avoided in the data analysis. Such biases have been shown to have significant impacts on the determined ratios in cases with low statistics ($^{129}$In) or overlapping peaks ($^{129}$Sn). The homogeneity of the MCP efficiency is reproduced with the GP and is implemented in the analysis routine. The efficiency correction reduces the deviation between the IYRs from multiple measurements of the same pair. However, the correction seems to be insufficient to eliminate all discrepancies and further investigations into the homogeneity of the MCP are necessary.

The decay of fission products and the integral feeding from the precursors in every step are analytically estimated with only few assumptions and initial input from the literature. The isomeric yield ratio of short-lived fission products is obtained from the observed counts on the MCP detector using analytical equations.

## Data Availability

This manuscript has associated data in a data repository. [Authors’ comment: All data included in this manuscript are available upon request by contacting with the corresponding author.]
